# Serum 25-Hydroxyvitamin D Level and Influenza Vaccine Immunogenicity in Children and Adolescents

**DOI:** 10.1371/journal.pone.0083553

**Published:** 2014-01-10

**Authors:** Michelle Science, Jonathon L. Maguire, Margaret L. Russell, Marek Smieja, Stephen D. Walter, Mark Loeb

**Affiliations:** 1 Division of Infectious Diseases, The Hospital for Sick Children, Toronto, Ontario, Canada; 2 Department of Clinical Epidemiology and Biostatistics, McMaster University, Hamilton, Ontario, Canada; 3 Applied Health Research Centre, Li Ka Shing Knowledge Institute of St. Michael's Hospital, Toronto, Ontario, Canada; 4 Department of Paediatrics, St Michael's Hospital, Toronto, Ontario, Canada; 5 Institute for Health Policy, Management and Evaluation, University of Toronto, Toronto, Ontario, Canada; 6 Department of Pediatrics, University of Toronto, Toronto, Ontario, Canada; 7 Department of Community Health Sciences, University of Calgary, Calgary, Alberta, Canada; 8 Department of Pathology and Molecular Medicine, McMaster University, Hamilton, Ontario, Canada; 9 Michael G DeGroote Institute for Infectious Disease Research, McMaster University, Hamilton, Ontario, Canada; Centers for Disease Control and Prevention, United States of America

## Abstract

**Background:**

Vaccination is an important strategy in the prevention of influenza, but immunologic response to vaccination can vary widely. Recent studies have shown an association between serum 25-hydroxyvitamin D (25[OH]D) levels and immune function. The purpose of this study was to determine if serum 25(OH)D level correlates with influenza vaccine immunogenicity in children and adolescents.

**Methods:**

We conducted a prospective cohort study of children age 3 to 15 years of age vaccinated with trivalent influenza vaccine (A/Brisbane/59/2007[H1N1]-like virus, A/Brisbane/10/2007 [H3N2]-like virus and B/Florida/4/2006-like virus) in Hutterite communities in Alberta, Saskatchewan and Manitoba. Serum 25(OH)D levels were measured at baseline and immunogenicity was assessed using hemagluttination inhibition (HAI) titers done at baseline and 3–5 weeks post vaccination. Logistic regression was used to assess the relationship between serum 25(OH)D level as both a continuous and dichotomous variable and seroprotection, seroconversion, fold increase in geometric mean titer (GMT) and post vaccination titer.

**Results:**

A total of 391 children and adolescents were included in the study and 221 (57% had post-vaccination HAI titers. The median serum 25(OH)D level was 61.0 nmol/L (Interquartile range [IQR] 50.0, 71.0). No relationship was found between serum 25(OH)D level and seroprotection (post-vaccination titer ≥40 and ≥320) or seroconversion (post-vaccination titer ≥40 for participants with pre-vaccine titer <10 or four-fold rise in post-vaccination titer for those with a pre-vaccine titer ≥10).

**Conclusion:**

Serum 25(OH)D level was not associated with influenza vaccine immunogenicity in otherwise healthy children and adolescents. Other strategies to enhance influenza vaccine response should continue to be evaluated in this population.

The role of serum 25(OH)D level in vaccine responsiveness in other populations, especially those hyporesponsive to influenza vaccination, requires further study.

## Introduction

Influenza is the cause of annual seasonal epidemics estimated to affect 5% to 10% of the population, posing an important public health burden [1]. Vaccination with influenza vaccine can reduce morbidity and mortality associated with influenza. Therefore, vaccination is a key strategy to prevent illness and transmission.

Protection obtained from influenza vaccination can vary widely depending on the vaccine match with circulating strains of influenza and the individual immune response [2]. The response is often reduced in immunocompromised patients, elderly patients, patients with chronic conditions and other individuals because of immunological changes of which many have yet to be fully elucidated [3]. Therefore, strategies to boost the effectiveness and duration of immunity after vaccination are of specific interest. Although strategies such as an increase in vaccine dose, intradermal injection, and use of adjuvants have been shown to increase immunogenicity [4]–[6], little is known about the effect of vitamins on influenza vaccine antibody response.

Recent evidence has shown that vitamin D is associated with both innate [7]–[9] and adaptive [10]–[12] immune responses and therefore may have a role in vaccine immunogenicity. However, the link between vitamin D levels and vaccine responsiveness remains theoretical and has received little focus in existing studies. Evidence to date includes mouse models which demonstrate that mucosal and systemic antibody responses to influenza are enhanced when the vaccine is co-administered with calcitriol (1,25-dihydroxyvitamin D) [13], [14]. However, a study in healthy adults failed to demonstrate a benefit from calcitriol intramuscular co-administration with the vaccine [15]. Furthermore, post-hoc analysis of a prospective influenza vaccine trial in HIV positive individuals did not find a difference in vaccine responsiveness between those receiving routine vitamin D supplementation and those not on supplementation [16]. These studies were limited by their lack of baseline serum 25(OH)D levels, making it uncertain as to whether individuals had low serum 25(OH)D levels at baseline prior to supplementation. More recently, a small study of 35 patients with prostate cancer found an association between baseline vitamin D level and influenza vaccine response [17].

Children are an important potential target group to optimize vaccine response given their potential role in transmission to high-risk groups [18]. This is also a group where vitamin D deficiency can be common [19]–[21].

The purpose of this study was to determine if there is an association between baseline serum 25-hydroxyvitamin D [25(OH)D] level and inactivated trivalent influenza vaccine immunogenicity in children and adolescents.

## Methods

### Study Design

A prospective cohort of children and adolescents were given trivalent influenza vaccine as part of a cluster randomized controlled trial (RCT) evaluating the effect of influenza vaccination on infection rates in Hutterite communities (clinicaltrials.gov: NCT00877396; isrctn.org: ISRCTN15363571) [18]. The research protocol was approved by McMaster University Research Ethics Review Board. Study subjects included children and adolescents age 3 to 15 years. Participants were randomly assigned by colony (n = 46) to receive either inactivated seasonal influenza vaccine or hepatitis A vaccine. Research nurses monitored participants for influenza infection with twice weekly assessments during the influenza season and infection was confirmed by a positive nasopharyngeal polymerase chain reaction (PCR) result. Blood specimens for serum 25(OH)D levels were collected at baseline. Serum from venous blood was frozen until batched analysis was performed according to the manufacturer's instructions using the DiaSorin LIAISON® chemiluminescence assay. Blood specimens for influenza antibody titers were collected at baseline and at least 3–5 weeks after vaccination. The paired samples were then tested using hemaggluttination inhibition (HAI) using turkey erythrocytes and reference antigens a for A/Brisbane/59/2007[H1N1]-like virus, A/Brisbane/10/2007 [H3N2]-like virus and B/Brisbane/60/2008-like virus which were the circulating strains [22]. Testing for B/Florida/4/2006-like virus was not conducted in the original RCT and therefore not available.

Only children and adolescents who received influenza vaccination and had vitamin D levels were included in the analysis. Covariables of interest included age, sex, presence of underlying comorbidities assessed by interview (heart/lung disease (including asthma), blood disorder, swallowing/choking disorder, ASA use, chronic metabolic condition, kidney/liver disease, immunodeficiency) and 25-hydroxyvitamin D level (nmol/L).

### Vaccination

Of the 743 children and adolescents with serum 25(OH)D levels included in the trial, 391 (53%) were randomized to receive influenza vaccination. The trivalent influenza vaccine (Vaxigrip; Sanofi Pasteur, Lyon France) contained purified surface antigen from the strains recommended for the 2008–2009 influenza season by the World Health Organization for the Northern Hemisphere: A/Brisbane/59/2007[H1N1]-like virus, A/Brisbane/10/2007 [H3N2]-like virus and B/Florida/4/2006-like virus. The 0.5 mL dose of vaccine included 15 µg of hemagluttinin antigen per recommended strain. Vaccine administration start dates ranged from October 30, 2008 for communities in Alberta to November 13, 2008 for communities in Manitoba. Participants received 0.5 mL of the influenza vaccine intramuscularly. Children less than 9 years of age with no history of previous influenza vaccine received a second dose of vaccine 4 weeks after the first dose, in keeping with influenza vaccination recommendations.

### Outcomes

The primary outcome was immunogenicity, using criteria that included seroprotection (post-vaccination titer ≥40) and seroconversion (post-vaccination titer ≥40 for participants with pre-vaccine titer <10 or four-fold rise in post-vaccination titer for those with a pre-vaccine titer ≥10), as defined by the Food and Drug Association (FDA) [23] and the Committee for Proprietary Medicinal Products (CPMP) [24]. We also explored the impact of defining seroprotection as a post-vaccination titer ≥320, a cut-point that may be more appropriate for children [25]. Other outcomes measured included change in antibody titer (four-fold change and fold increase in geometric mean titers [GMT]) and antibody level post vaccination (log_2_-transformed). Fold increase in GMT was calculated by determining the ratio of raw post-vaccination to pre-vaccination titer, calculating the arithmetic mean of the log of these ratios and calculating the exponent and associated 95% confidence interval.

### Statistical Analysis

Baseline characteristics were described using mean and standard deviation for normally distributed data and median and interquartile range (IQR) for non-normal distributions. Characteristics were compared between those who had serology and those missing serology using the independent t-test or Wilcoxon rank sum test for continuous variables and chi-square or Fisher's exact test for dichotomous outcomes.

For each outcome, analyses were conducted on the antibody titers to each influenza A antigen individually (A/Brisbane/59/2007[H1N1]-like virus and A/Brisbane/10/2007[H3N2]-like virus). B/Brisbane/60/2008-like virus antibody titers were also analyzed despite the different vaccine antigen (B/Florida/4/2006-like virus) to assess for potential cross-protection between lineages.

Logistic regression was used to examine serum 25(OH)D level as a predictor of dichotomous outcomes (seroprotection, seroconversion and four-fold change in antibody titers). All relevant covariables (age, sex, presence of at least one comorbidity, serum 25(OH)D level) were first evaluated using univariable logistic regression. Vitamin D levels were analyzed as both a continuous variable (log transformed to correct positive skew) and dichotomized based on the American Academy of Pediatrics (AAP)(<50 nmol/L) and Canadian Pediatric Society (CPS)(<75 nmol/L) recommendations. Variables with a *p* value<0.1 were considered for inclusion in the multivariable model and the final model was determined using a step-wise backwards elimination method. It was decided *a priori* to adjust the final model for age and sex and to include serum 25(OH)D as a continuous variable.

Linear regression was used to examine the relationship between covariables and post vaccination HAI titers. Titers were log_2_-transformed using titer_(transformed)_ = log_2_(titer/5) resulting in the following: 0 = no HAI activity, 1 = 1∶10, 2 = 1∶20, 3 = 1∶40 etc. Covariables were analyzed and included in the final model as outlined for the logistic regression model. Generalized estimating equations (GEE) were used to account for clustering at the colony level for both regression analyses.

Geometric mean titers (GMT) were calculated at baseline and post vaccination and compared between subjects grouped by serum 25(OH)D levels based on the AAP (<50 nmol/L) and CPS (<75 nmol/L) cut-offs. Fold increase in GMT was calculated in each group (ratio of GMT pre and post vaccination) and the arithmetic mean of the log of the fold increase was compared using the independent t-test.

All estimates are presented with 95% confidence intervals. A *p* value<0.05 was considered significant. SPSS version 20 (SPSS Inc, Chicago, IL) was used to conduct the analyses.

## Results

Baseline characteristics are summarized in Table 1. A total of 391 children from 21 communities received influenza vaccination; 221 (57%) had post-vaccination serology and could be included in the immunogenicity analyses. There were no significant differences between groups based on availability of serology aside from sex (Table 1). The mean age was 9.2 years (standard deviation [SD] 3.3); 48% were male. The median serum 25(OH)D level was 61.0 nmol/L (IQR 50.0, 71.0). Serum 25(OH)D levels were taken between October 16, 2008 and January 29, 2009. Most sera were drawn between October and December 2008 (97%); no samples were taken after the post-vaccination titers. Post-vaccination titers were drawn a median of 11 weeks after the initial titer (IQR 6.9, 21.3).

**Table 1:** Baseline Characteristics of Participants.

	Overall*	Serology Available	No Serology	p-value**
	(n = 391)	(n = 221)	(n = 170)	
**Age**, Mean (SD, Range)	9.26 (3.39, 3–15)	9.16 (3.28, 3–15)	9.39 (3.42, 3–15)	0.506
**Male**	169 (43.2%)	106 (48.0%)	63 (37.1%)	0.031
**Comorbidities**				
**> = 1 Comorbidity**	12 (3.1%)	10 (4.5%)	2 (1.2%)	0.057
**Asthma**	8 (2.0%)	7 (3.2%)	1 (0.6%)	0.072
**Vitamin D Level (nmol/L)**, Median (IQR)	61.0 (51.0, 72.0)	61.0 (50.0, 71.0)	62.0 (53.0, 74.0)	0.181
**Vitamin D Deficiency**				
**<25 nmol/L**	3 (0.8%)	2 (0.9%)	1 (0.6%)	0.598
**AAP<50 nmol/L**	82 (21%)	54 (24.4%)	28 (16.5%)	0.055
**CPS<75 nmol/L**	305 (78%)	176 (79.6%)	129 (75.9%)	0.374

Categorical variables presented as number (%).

Independent t-test or Wilcoxon rank sum test for continuous variables, chi-square or Fisher's exact test for dichotomous variables.

The numbers and proportions of individuals with each outcome are summarized in table 2. Post vaccination titers were greater than or equal to 40 (seroprotection) for A/Brisbane/10/2007[H3N2] in 159 (71.9%) participants, A/Brisbane/59/2007[H1N1] in 138 (62.4%) and both antigens in 127 (57.5%) participants. Seroprotection against B/Brisbane/60/2008 occurred in 79 (35.7%) participants. When using a cut-off of 1∶320, seroprotection rates were lower occurring in 48.1% (A/Brisbane/10/2007[H3N2]), 38.3% (A/Brisbane/59/2007[H1N1]), 17.2% (both A) and 8.9% (B/Brisbane/60/2008) of participants.

**Table 2:** Summary of Outcomes by Influenza Strain.

	Number (percentage)
	n = 221
**Number with Pre-Vaccination titer > = 1∶40**	
A/Brisbane/10/2007[H3N2]	157 (71.0)
A/Brisbane/59/2007[H1N1]	93 (42.1)
B/Brisbane/60/2008	33 (14.9)
**Seroprotection (Number with Post-Vaccination titer > = 1∶40)**	
A/Brisbane/10/2007[H3N2]	159 (71.9)
A/Brisbane/59/2007[H1N1]	138 (62.4)
Both A strains	127 (57.5)
B/Brisbane/60/2008	79 (35.7)
**Seroprotection (Number with Post-Vaccination titer > = 1∶320)**	
A/Brisbane/10/2007[H3N2]	114 (48.1)
A/Brisbane/59/2007[H1N1]	92 (38.3)
Both A strains	66 (17.2)
B/Brisbane/60/2008	34 (8.9)
**Seroconversion (Pre-titer <10 and post > = 40)**	
A/Brisbane/10/2007[H3N2]	50 (22.6)
A/Brisbane/59/2007[H1N1]	76 (34.4)
Both A strains	24 (10.9)
B/Brisbane/60/2008	49 (22.2)
**Seroconversion (Pre titer > = 10 and four-fold change)**	
A/Brisbane/10/2007[H3N2]	44 (19.9)
A/Brisbane/59/2007[H1N1]	23 (23.1)
Both A strains	15 (6.8)
B/Brisbane/60/2008	17 (7.7)
**Seroconversion (Pre-titer <10 and post > = 40 OR Pre titer > = 10 and four-fold change)**	
A/Brisbane/10/2007[H3N2]	94 (42.5)
A/Brisbane/59/2007[H1N1]	127 (57.5)
Both A strains	77 (34.8)
B/Brisbane/60/2008	66 (29.9)
**Four-Fold Change in Titer**	
A/Brisbane/10/2007[H3N2]	96 (43.4)
A/Brisbane/59/2007[H1N1]	128 (57.9)
Both A strains	79 (35.7)
B/Brisbane/60/2008	73 (33)

In univariable analysis, there was no significant association between serum 25(OH)D level and seroprotection against any strain using a cut-off of 1∶40 (Table 3). However, the point estimates did suggest a relationship between higher serum 25(OH)D level and increased odds of seroprotection to both A/Brisbane/10/2007[H3N2] (OR 1.52, 95% CI 0.69, 3.34, p = 0.295) and A/Brisbane/59/2007[H1N1] (OR 1.42, 95% CI 0.52, 3.91, p = 0.587). Similarly, the point estimates suggested decreased odds of seroprotection to all three strains if serum 25(OH)D levels were lower than 50 nmol/L and lower than 75 nmol/L, but the estimates were not statistically significant. There was also no significant association found using a cut-off of 1∶320.

**Table 3:** Predictors of Seroprotection (Titer ≥1∶40) after Influenza Vaccination by Influenza Strain (Univariable Analyses).

	Seroprotection (Post Vaccination Antibody Titer ≥1∶40)					
	A/Brisbane/10/2007		A/Brisbane/59/2007		B/Brisbane/60/2008	
	[H3N2]		[H1N1]			
	OR (95% CI)	p-value	OR (95% CI)	p-value	OR (95% CI)	p-value
**Age (per 1 year change)**	0.94 (0.86 1.02)	0.128	0.96 (0.87, 1.06)	0.389	0.99 (0.86, 1.15)	0.930
**<5 years**	0.58 (0.27, 1.25)	0.166	0.61 (0.20, 1.82)	0.606	0.92 (0.26, 3.23)	0.893
**5–9 years**	0.63 (0.37, 1.07)	0.088	0.67 (0.34, 1.28)	0.665	1.23 (0.60, 2.50)	0.571
**>9 years**	Reference					
**Male**	1.17 (0.55, 2.49)	0.685	0.57 (0.29, 1.12)	0.104	0.93 (0.44, 1.98)	0.855
**Comorbidity**	1.59 (0.49, 5.20)	0.443	0.59 (0.26, 1.33)	0.202	0.19 (0.03, 1.42)	0.189
**Log serum 25(OH)D**	1.52 (0.69, 3.34)	0.295	1.42 (0.52, 3.91)	0.495	0.85 (0.29, 2.54)	0.771
**Vitamin D Deficiency**						
**AAP<50**	0.72 (0.48, 1.06)	0.097	0.84 (0.44, 1.59)	0.587	0.97 (0.41, 2.31)	0.942
**CPS<75**	0.92 (0.50, 1.70)	0.781	0.79 (0.34, 1.83)	0.589	0.80 (0.47, 1.34)	0.392

Seroconversion occurred in 94 (42.5%) children in response to A/Brisbane/10/2007[H3N2], 127 (57.5%) in response to A/Brisbane/59/2007[H1N1] and 66 (29.9%) to B/Brisbane/60/2008. In univariable and multivariable analysis, the presence of a comorbidity resulted in reduced odds of seroconversion (OR 0.17, 95% CI 0.03, 0.88, p = 0.034) to A/Brisbane/59/2007[H1N1] but not to the other strains. None of the other covariables, including serum 25(OH)D, were associated with seroconversion to any of the strains (Table 4). Similarly, there was no association between any of the covariables and presence of a four-fold change in titer or post-vaccination titer level (log_2_transformed).

**Table 4:** Predictors of Seroconversion after Influenza Vaccination by Influenza Strain (Univariable Analyses).

	Seroconversion*					
	A/Brisbane/10/2007		A/Brisbane/59/2007		B/Brisbane/60/2008	
	[H3N2]		[H1N1]			
	OR (95% CI)	p-value	OR (95% CI)	p-value	OR (95% CI)	p-value
**Age (per 1 year change)**	0.99 (0.89, 1.12)	0.933	0.97 (0.88, 1.07)	0.502	0.97 (0.86, 1.09)	0.632
**<5 years**	0.68 (0.27, 1.76)	0.684	0.61 (0.18, 2.04)	0.419	0.71 (0.21, 2.41)	0.233
**5–9 years**	1.16 (0.60, 2.26)	0.666	0.69 (0.37, 1.31)	0.261	1.43 (0.80, 2.56)	0.233
**>9 years**	Reference					
**Male**	1.34 (0.68, 2.62)	0.396	0.60 (0.29, 1.23)	0.164	0.95 (0.45, 1.99)	0.881
**Comorbidity**	0.90 (0.39, 2.04)	0.794	0.17 (0.03, 0.88)	0.034	**	
**Log serum 25(OH)D**	1.02 (0.54, 1.92)	0.951	0.98 (0.44, 2.15)	0.952	0.78 (0.21, 2.92)	0.706
**Vitamin D Deficiency**						
**AAP<50**	1.00 (0.60, 1.67)	0.990	0.82 (0.54, 1.24)	0.346	1.11 (0.42, 2.90)	0.837
**CPS<75**	1.14 (0.63, 2.07)	0.668	1.38 (0.87, 2.20)	0.173	0.93 (0.48, 1.81)	0.827

post-vaccination titer ≥1∶40 for participants with pre-vaccine titer <1∶10 and four-fold rise in post-vaccination titer for those with a pre-vaccine titer ≥1∶10.

unable to estimate because of Hessian matrix singularity.

The fold increase in GMT was highest for A/Brisbane/59/2007[H1N1] (mean 5.25, 95% CI 3.39, 8.15) compared to A/Brisbane/10/2007[H3N2] (mean 1.39, 95% CI 0.37, 2,15). The fold increase in GMT for B/Brisbane/60/2008 was 2.21 (95%CI 1.75, 2.79). There was no significant difference in fold change in GMT between groups based on vitamin D level cut-offs of 50 nmol/l and 75 nmol/L (Table 5).

**Table 5:** Geometric Mean Titers (baseline, post-vaccination and fold change) by Influenza Strain and comparison based on serum 25-hydroxyvitamin D level.

	Overall	Serum 25(OH)D Level			Serum 25(OH)D Level		
		<50 nmol/L	> = 50 nmol/L	p-value*	<75 nmol/L	> = 75 nmol/L	p-value*
**A/Brisbane/10/2007[H3N2]**							
Baseline	93.9	114.6	88.0	0.443	91.5	103.9	0.727
(95% CI)	(70.2, 125.5)	(62.6, 209.8)	(63.0,122.9)		(65.0, 128.6)	(61.3, 176.2)	
Post	130.9	123.8	133.3	0.839	133.0	123.1	0.843
(95% CI)	(96.3, 178.0)	(63.1, 242.8)	(94.2, 188.7)		(94.1, 187.9)	(61.3, 247.2)	
**A/Brisbane/59/2007[H1N1]**							
Baseline	22.8	20.8	23.5	0.647	19.9	38.8	0.020
(95% CI)	(18.2, 28.6)	(12.6, 34.4)	(18.2, 30.4)		(15.5, 25.6)	(22.7, 66.2)	
Post	119.9	116.1	121.2	0.918	112.2	155.1	0.465
(95% CI)	(84.4, 170.3)	(53.9, 250.2)	(81.4, 180.2)		(75.5, 166.8)	(71.0, 339.0)	
**B/Brisbane/60/2008**							
Baseline	8.9	7.3	9.5	0.169	8.4	11.0	0.181
(95% CI)	(7.6, 10.4)	(5.6, 9.6)	(7.9, 11.4)		(7.2, 0.9)	(7.1, 16.9)	
Post	19.7	18.9	20.0	0.859	19.1	22.6	0.581
(95% CI)	(15.5, 25.2)	(11.7, 30.8)	(15.0, 26.6)		(14.4, 25.2)	(13.4, 38.3)	
**Fold Increase GMT**							
H3N2	1.39	1.08	1.51	0.509	1.45	1.18	0.708
(95% CI)	(0.91, 2.15)	(0.43, 2.74)	(0.93, 2.48)		(0.88, 2.41)	(0.52, 2.68)	
H1N1	5.25	5.58	5.15	0.877	5.63	4.00	0.537
(95% CI)	(3.39, 8.15)	(2.05, 15.23)	(3.16, 8.40)		(3.41, 9.30)	(1.58, 10.12)	
Influenza B	2.21	2.59	2.10	0.452	2.25	2.06	0.764
(95% CI)	(1.75, 2.79)	(1.69, 3.96)	(1.59, 2.77)		(1.73, 2.94)	(1.26, 3.41)	

t-test comparing means of log titers between serum 25(OH)D level groups.

### Sensitivity Analyses

Given the range in timing of post-vaccination titers (median 11 wks) and to account for possible waning immunity with time, a sensitivity analysis was conducted excluding subjects that had post-vaccination titers more than 3 months after vaccination; this group also excluded the subjects that had late serum 25(OH)D levels. In this analysis, there was no change in the significance of covariables for any of the outcomes. Additionally, the analyses were conducted including the time from last vaccine dose as a variable in the analysis. This did not change the results appreciably.

In a second sensitivity analysis, participants with proven influenza infection (n = 22) were excluded given the probability that the antibody change was related to natural infection not vaccine response. There was no appreciable change in the results. We also excluded participants who had titers done after the start of the influenza season (December 28, 2008). There was no association between vitamin D and seroprotection or seroconversion.

In order to account for pre-vaccination seroprotection, participants with baseline titers ≥40 were excluded from the analysis. There was no association found between vitamin D and seroprotection.

Finally, participants who may not have had enough time to respond to the vaccine (less than 2 weeks between vaccine and serology) or were not appropriately vaccinated (i.e. only 1 vaccine dose in participants less than 9 years of age) were excluded (n = 11). There was no appreciable change in the results.

## Discussion

The main finding of this study was that serum 25(OH)D level was not significantly associated with immunogenicity as measured by seroprotection (post-vaccination titer ≥40 or ≥320) and seroconversion. Serum 25(OH)D level was also not associated with other commonly reported measures of vaccine immunogenicity, including presence of a four-fold rise in antibody titer, the fold change in GMT or post vaccination titer.

This finding is consistent with three randomized controlled trials of vitamin D supplementation in influenza vaccinated subjects, one in healthy adults [15], one in children [26] and the other in HIV-infected adults [16]. All three studies found no effect of supplementation on serologic responses. It is also consistent with a recent prospective cohort study in adults [27]. We also did not find an association between serum 25(OH)D level and any of the commonly used immunogenicity criteria. However, this failure to detect an association may be related to sample size and power.

Our findings are different from a recent study of influenza vaccination in prostate cancer patients (n = 35) which found that significantly more participants with a replete vitamin D status, defined as the upper quartile of serum 25(OH)D levels, had titers ≥1∶40 at 3 months against any of the 3 strains [17]. They also reported an association between serum 25(OH)D levels and serologic response (p = 0.0446), but the magnitude of effect and confidence intervals were not provided. This trial was limited by the definition of serologic response (response to any of the three antigens) and the high serum 25(OH)D levels in the population (median 44.8 ng/mL≈112 nmol/L). Another possible explanation for the divergent results is the different patient population. The effect of serum 25(OH)D level on vaccine immunogenicity may be different in the immunocompromised population, a group which tends to be more hyporesponsive to influenza vaccinations [3].

Overall, seroprotection and seroconversion proportions for the Influenza A strains were in keeping with the recommended standards [28]. A better seroconversion percentage and fold change in GMT were noted for the A/Brisbane/59/2007[H1N1] strain compared to the A/Brisbane/10/2007[H3N2] strain and this was reflected in the protective efficacy with 8 confirmed cases of H3N2, but no confirmed cases of seasonal H1N1.

With respect to the Influenza B immunogenicity data, another finding in our study was the rates of seroprotection and seroconversion to B/Brisbane/60/2008-like virus despite vaccination with B/Florida/4/2006-like virus. These strains are of different lineages, B/Victoria and B/Yamagata respectively, and are believed to result in very little cross-protection [2]. However, we observed seroprotection and seroconversion percentages of 35% and 30%, respectively to B/Brisbane/60/2008-like virus. Only 2 subjects who had seroconversion had proven influenza B infection, therefore these changes in antibody titer represent either vaccine response or sub-clinical infection. Although these percentages are lower than recommended for vaccine licensure (seroprotection >60%, seroconversion >30%) [28], it does suggest some potential cross-protection if the antibody responses are related to vaccination.

Limitations of this study include the variability in the timing of both serum 25(OH)D levels and the post-vaccination titers. However, a recent study in a cohort of urban children in Toronto, found the variability in vitamin D levels from winter to summer months was less than 10 nmol/L [29]. In addition, two separate sensitivity analyses, first excluding participants with levels drawn more than 3 months after baseline levels/vaccination (n = 72, 32%) and second including the time from last vaccine dose as a variable in the analysis, revealed similar results to our primary analysis. Secondly, antibody titers were only available on study participants who agreed to the follow-up bloodwork (n = 221, 57%). This sample size limitation may have impacted our ability to detect a significant difference. With this sample size and 80% power, the minimum hazard ratio that the study was powered to detect was 2.78, a hazard ratio well above a clinically meaningful effect. In addition, we did not have the specimens to measure functional cell-mediated immune response. We also acknowledge that previous influenza vaccine history may have influenced the outcome of seroprotection. This was the reason for collecting baseline antibody levels and looking at multiple immunogenicity outcomes, including seroconversion. Finally, this study was conducted in Hutterite children and adolescents, which may limit generalizability to other populations.

In conclusion, we found that serum 25(OH)D level was not associated with immunogenicity in children and adolescents. However, sample size may have limited our ability to detect a significant difference and larger studies are warranted. Given the important role of children and adolescents in the spread of viral infections and the potential benefits from their immunization to protect other higher risk groups, other strategies should continue be evaluated to enhance the vaccine immune response. The role of serum 25(OH)D level in influenza vaccine immunogenicity in other populations, specifically immunocompromised patients, may warrant further study.
